# CRISPR Gene Therapy: Applications, Limitations, and Implications for the Future

**DOI:** 10.3389/fonc.2020.01387

**Published:** 2020-08-07

**Authors:** Fathema Uddin, Charles M. Rudin, Triparna Sen

**Affiliations:** ^1^Department of Medicine, Thoracic Oncology Service, Memorial Sloan Kettering Cancer Center, New York, NY, United States; ^2^Weill Cornell Medicine, Cornell University, New York, NY, United States

**Keywords:** gene therapy, CRISPR/Cas9, homology-directed repair (HDR), non-homologous end joining (NHEJ), clinical trial, ethics

## Abstract

A series of recent discoveries harnessing the adaptive immune system of prokaryotes to perform targeted genome editing is having a transformative influence across the biological sciences. The discovery of Clustered Regularly Interspaced Short Palindromic Repeats (CRISPR) and CRISPR-associated (Cas) proteins has expanded the applications of genetic research in thousands of laboratories across the globe and is redefining our approach to gene therapy. Traditional gene therapy has raised some concerns, as its reliance on viral vector delivery of therapeutic transgenes can cause both insertional oncogenesis and immunogenic toxicity. While viral vectors remain a key delivery vehicle, CRISPR technology provides a relatively simple and efficient alternative for site-specific gene editing, obliviating some concerns raised by traditional gene therapy. Although it has apparent advantages, CRISPR/Cas9 brings its own set of limitations which must be addressed for safe and efficient clinical translation. This review focuses on the evolution of gene therapy and the role of CRISPR in shifting the gene therapy paradigm. We review the emerging data of recent gene therapy trials and consider the best strategy to move forward with this powerful but still relatively new technology.

## Introduction

Gene therapy as a strategy to provide therapeutic benefit includes modifying genes via disruption, correction, or replacement (1). Gene therapy has witnessed both early successes and tragic failures in a clinical setting. The discovery and development of the CRISPR/Cas9 system has provided a second opportunity for gene therapy to recover from its stigma and prove to be valuable therapeutic strategy. The recent advent of CRISPR technology in clinical trials has paved way for the new era of CRISPR gene therapy to emerge. However, there are several technical and ethical considerations that need addressing when considering its use for patient care. This review aims to (1) provide a brief history of gene therapy prior to CRISPR and discuss its ethical dilemmas, (2) describe the mechanisms by which CRISPR/Cas9 induces gene edits, (3) discuss the current limitations and advancements made for CRISPR technology for therapeutic translation, and (4) highlight a few recent clinical trials utilizing CRISPR gene therapy while opening a discussion for the ethical barriers that these and future trials may hinge upon.

## Gene Therapy Prior to Crispr—History, Hurdles, and its Future

### Origins of Gene Therapy

The introduction of gene therapy into the clinic provided hope for thousands of patients with genetic diseases and limited treatment options. Initially, gene therapy utilized viral vector delivery of therapeutic transgenes for cancer treatment (2) or monogenic disease (3). One of these pioneering clinical trials involved *ex vivo* retroviral delivery of a selective neomycin-resistance marker to tumor infiltrating leukocytes (TILs) extracted from advanced melanoma patients (4). Although the neomycin tagging of TILs did not have a direct therapeutic intent and was used for tracking purposes, this study was the first to provide evidence for both the feasibility and safety of viral-mediated gene therapy. Soon after, the first clinical trial that used gene therapy for therapeutic intent was approved in 1990 for the monogenic disease adenosine deaminase-severe combined immunodeficiency (ADA-SCID). Two young girls with ADA-SCID were treated with retroviruses for *ex vivo* delivery of a wildtype adenosine deaminase gene to autologous T-lymphocytes, which were then infused back into the patients (5, 6). While one patient showed moderate improvement, the other did not (5, 6) Although initial results were suboptimal, the early evidence of feasibility prompted multiple subsequent gene therapy trials using viral-mediated gene edition. However, this was followed by some major setbacks.

### Tragic Setbacks for Gene Therapy

Jesse Gelsinger, an 18-year-old with a mild form of the genetic disease ornithine transcarbamylase (OTC) deficiency, participated in a clinical trial which delivered a non-mutated OTC gene to the liver through a hepatic artery injection of the recombinant adenoviral vector housing the therapeutic gene. Unfortunately, Jesse passed away 4 days after treatment (7). The adenovirus vector triggered a much stronger immune response in Jesse than it had in other patients, causing a chain of multiple organ failures that ultimately led to his death (8). At the time of the trial, adenoviral vectors were considered reasonably safe. In preclinical development, however, two of the rhesus monkeys treated with the therapy developed a similar pattern of fatal hepatocellular necrosis (9). Shortly after, another gene therapy trial led to the development of leukemia in several young children induced by insertional oncogenesis from the therapy (10). These trials opened for two forms of SCID (SCID-X1 or common ɤ chain deficiency) and adenosine deaminase deficiency (ADA). The therapy used ɤ-retroviral vectors for *ex vivo* delivery of therapeutic transgenes to autologous CD34+ hematopoietic stem cells, which were reintroduced to the patients (10). Five patients developed secondary therapy-related leukemia, one of whom died from the disease (11). Further investigation revealed integration of the therapeutic gene into the *LMO2* proto-oncogene locus, presumably resulting in the development of leukemia (12). Subsequent analyses have suggested a higher frequency of insertional mutagenesis events with ɤ-retroviral vectors relative to other vectors (13). Together, these tragic events prompted substantial *post-hoc* concerns regarding the nature of appropriate informed consent and the stringency of safety and eligibility parameters for gene therapy experimentation in humans (14).

### Shifting the Gene Therapy Paradigm

Almost two decades after these cases, gene therapy returned in clinical trials with reengineered viruses designed with safety in mind. Current clinical approaches are being scrutinized for evidence of insertional mutagenesis and adverse immunogenic reactions (15–18). Non-viral vectors have been used as an alternative method for gene delivery, which have reduced immunogenicity compared to their viral counterparts and therefore greater tolerance for repeated administration. A concern is whether these methods can be optimized to provide equivalent efficiency of gene delivery to that provided by viruses (19).

While viral vectors continue to be essential for current gene therapy, the concerns and limitations of viral-mediated gene edition has broadened the diversity of gene-editing approaches being considered. Rather than introducing the therapeutic gene into a novel (and potentially problematic) locus, a more attractive strategy would be to directly correct the existing genetic aberrations *in situ*. This alternative would allow the pathological mutation to be repaired while averting the risk of insertional oncogenesis. The discovery and repurposing of nucleases for programmable gene editing made this possible, beginning with the development of zinc finger nucleases (ZFN) (20, 21), followed by transcription activator-like effector nucleases (TALENs), meganucleases, and most recently, the CRISPR/Cas system (22). While the other gene-editing tools can induce genome editing at targeted sites under controlled conditions, the CRISPR/Cas system has largely supplanted these earlier advances due to its relatively low price, ease of use, and efficient and precise performance. However, this technology is often delivered with adeno-associated virus (AAV) vectors, and thus does not completely avert risks associated with viruses. Other delivery options are available to circumvent this issue, each with their own advantages and challenges (see Delivery of CRISPR Gene Therapy section). Of the CRISPR/Cas systems, CRISPR/Cas9 is the most developed and widely used tool for current genome editing.

## CRISPR/Cas9 Mediated Gene Editing

### Pioneering Discoveries in CRISPR/Cas9 Technology

**Figure 1:**
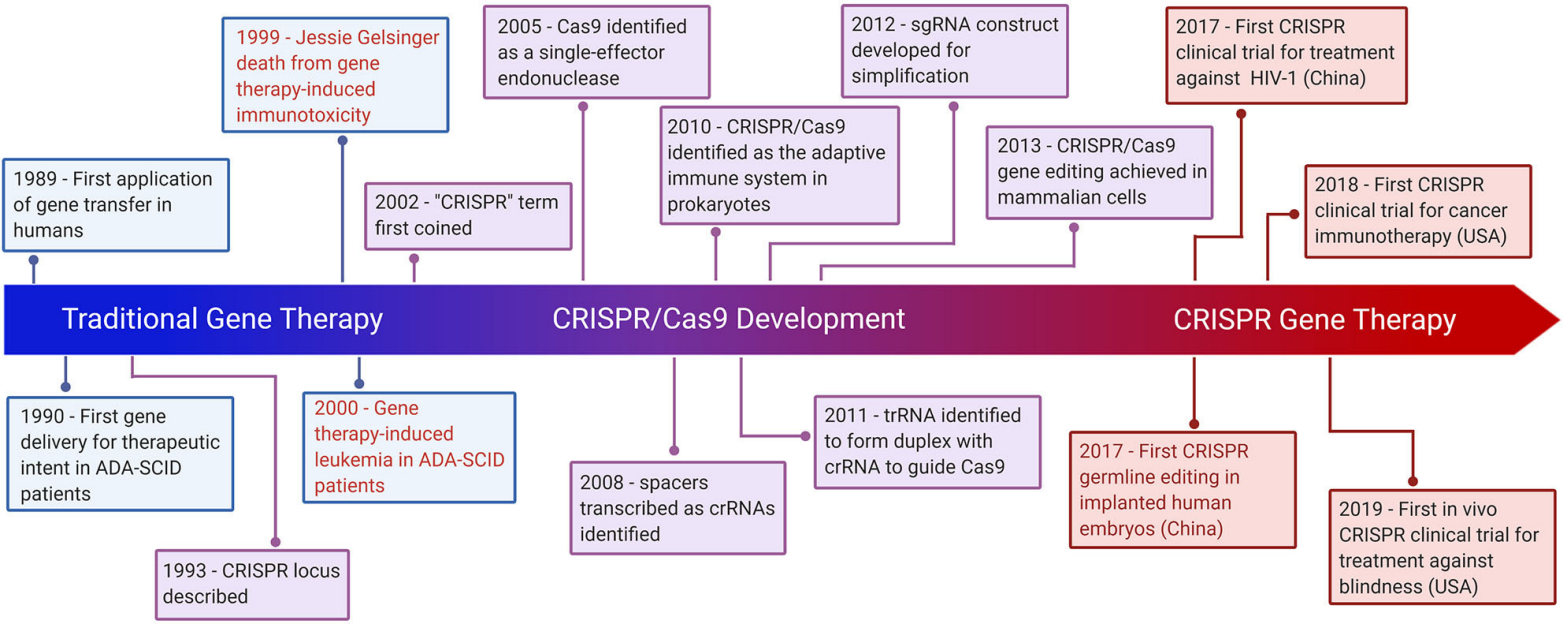
Hallmarks of CRISPR Gene Therapy. Timeline highlighting major events of traditional gene therapy, CRISPR development, and CRISPR gene therapy. The text in red denotes gene therapy events which have raised significant ethical concerns.

The bacterial CRISPR locus was first described by Francisco Mojica (23) and later identified as a key element in the adaptive immune system in prokaryotes (24). The locus consists of snippets of viral or plasmid DNA that previously infected the microbe (later termed “spacers”), which were found between an array of short palindromic repeat sequences. Later, Alexander Bolotin discovered the Cas9 protein in *Streptococcus thermophilus*, which unlike other known Cas genes, Cas9 was a large gene that encoded for a single-effector protein with nuclease activity (25). They further noted a common sequence in the target DNA adjacent to the spacer, later known as the protospacer adjacent motif (PAM)—the sequence needed for Cas9 to recognize and bind its target DNA (25). Later studies reported that spacers were transcribed to CRISPR RNAs (crRNAs) that guide the Cas proteins to the target site of DNA (26). Following studies discovered the trans-activating CRISPR RNA (tracrRNA), which forms a duplex with crRNA that together guide Cas9 to its target DNA (27). The potential use of this system was simplified by introducing a synthetic combined crRNA and tracrRNA construct called a single-guide RNA (sgRNA) (28). This was followed by studies demonstrating successful genome editing by CRISPR/Cas9 in mammalian cells, thereby opening the possibility of implementing CRISPR/Cas9 in gene therapy (29) (Figure 1).

### Mechanistic Overview of CRISPR/Cas9-Mediated Genome Editing

**Figure 2:**
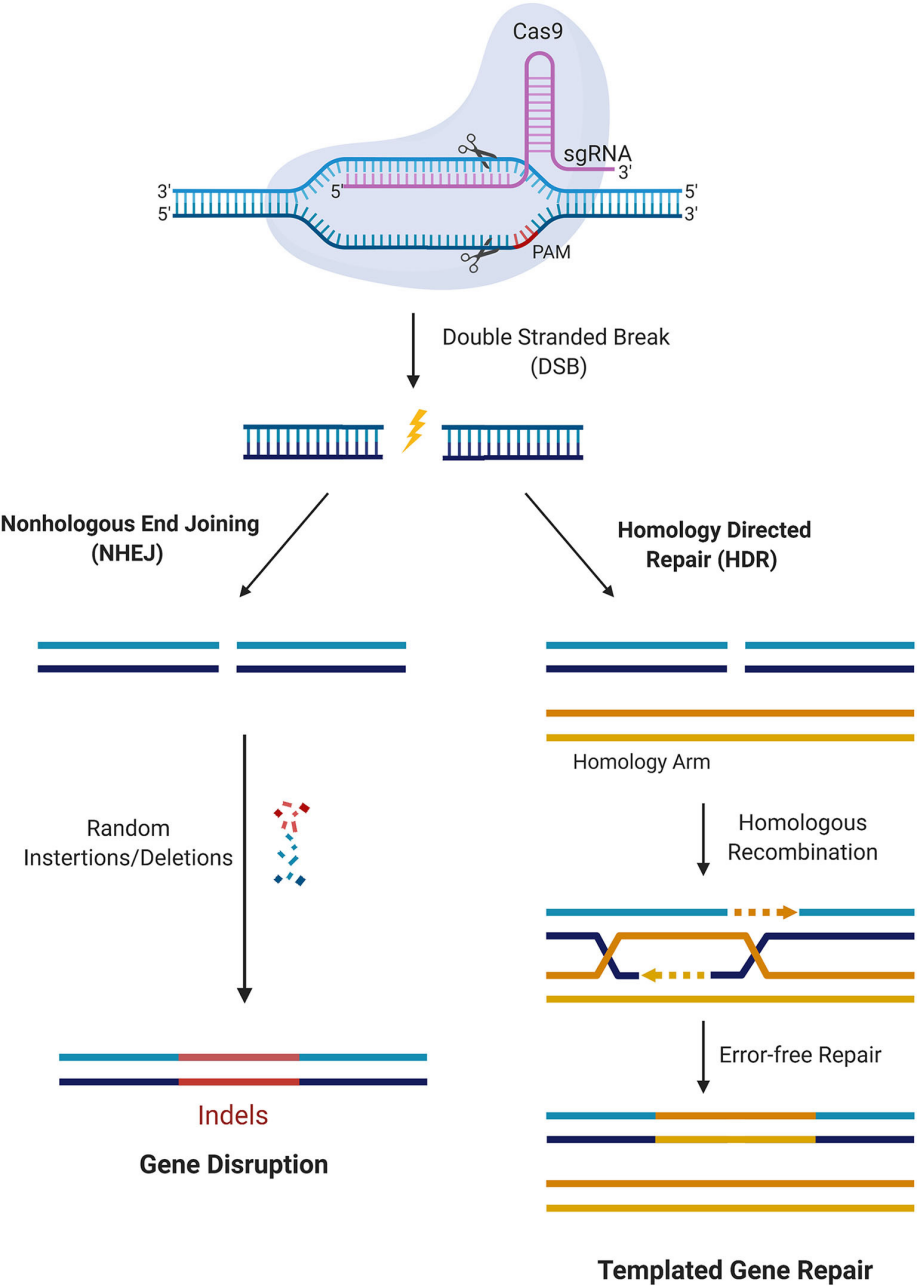
CRISPR/Cas9 mediated gene editing. Cas9 in complex with the sgRNA targets the respective gene and creates DSBs near the PAM region. DNA damage repair proceeds either through the NHEJ pathway or HDR. In the NHEJ pathway, random insertions and deletions (indels) are introduced at the cut side and ligated resulting in error-prone repair. In the HDR pathway, the homologous chromosomal DNA serves as a template for the damaged DNA during repair, resulting in error-free repair.

CRISPR/Cas9 is a simple two-component system used for effective targeted gene editing. The first component is the single-effector Cas9 protein, which contains the endonuclease domains RuvC and HNH. RuvC cleaves the DNA strand non-complementary to the spacer sequence and HNH cleaves the complementary strand. Together, these domains generate double-stranded breaks (DSBs) in the target DNA. The second component of effective targeted gene editing is a single guide RNA (sgRNA) carrying a scaffold sequence which enables its anchoring to Cas9 and a 20 base pair spacer sequence complementary to the target gene and adjacent to the PAM sequence. This sgRNA guides the CRISPR/Cas9 complex to its intended genomic location. The editing system then relies on either of two endogenous DNA repair pathways: non-homologous end-joining (NHEJ) or homology-directed repair (HDR) (Figure 2). NHEJ occurs much more frequently in most cell types and involves random insertion and deletion of base pairs, or indels, at the cut site. This error-prone mechanism usually results in frameshift mutations, often creating a premature stop codon and/or a non-functional polypeptide. This pathway has been particularly useful in genetic knock-out experiments and functional genomic CRISPR screens, but it can also be useful in the clinic in the context where gene disruption provides a therapeutic opportunity. The other pathway, which is especially appealing to exploit for clinical purposes, is the error-free HDR pathway. This pathway involves using the homologous region of the unedited DNA strand as a template to correct the damaged DNA, resulting in error-free repair. Experimentally, this pathway can be exploited by providing an exogenous donor template with the CRISPR/Cas9 machinery to facilitate the desired edit into the genome (30).

## Limitations and Advancements of CRISPR/Cas9

### Off-Target Effects

A major concern for implementing CRISPR/Cas9 for gene therapy is the relatively high frequency of off-target effects (OTEs), which have been observed at a frequency of ≥50% (31). Current attempts at addressing this concern include engineered Cas9 variants that exhibit reduced OTE and optimizing guide designs. One strategy that minimizes OTEs utilizes Cas9 nickase (Cas9n), a variant that induces single-stranded breaks (SSBs), in combination with an sgRNA pair targeting both strands of the DNA at the intended location to produce the DSB (32). Researchers have also developed Cas9 variants that are specifically engineered to reduce OTEs while maintaining editing efficacy (Table 1). SpCas9-HF1 is one of these high-fidelity variants that exploits the “excess-energy” model which proposes that there is an excess affinity between Cas9 and target DNA which may be enabling OTEs. By introducing mutations to 4 residues involved in direct hydrogen bonding between Cas9 and the phosphate backbone of the target DNA, SpCas9-HF1 has been shown to possess no detectable off-target activity in comparison to wildtype SpCas9 (35). Other Cas9 variants that have been developed include evoCas9 and HiFiCas9, both of which contain altered amino acid residues in the Rec3 domain which is involved in nucleotide recognition. Desensitizing the Rec3 domain increases the dependence on specificity for the DNA:RNA heteroduplex to induce DSBs, thereby reducing OTEs while maintaining editing efficacy (38, 39). One of the more recent developments is the Cas9_R63A/Q768A variant, in which the R63A mutation destabilizes R-loop formation in the presence of mismatches and Q768A mutation increases sensitivity to PAM-distal mismatches (49). Despite the different strategies, the rational for generating many Cas9 variants with reduced OTEs has been to ultimately reduce general Cas9 and DNA interactions and give a stronger role for the DNA:RNA heteroduplex in facilitating the edits.

**Table 1:** Cas9 variants.

**Bacterial origin**	**Cas9 variant**	**Advantage**	**Variant mutation**	**PAM**	**References**
*Streptococcus pyogenes*	Cas9-D1135E	Improved PAM recognition	D1135E	NGG	(33)
	Cas9-VQR	Altered PAM	D1135V/R1335Q/T1337R	NGAN or NGNG	
	Cas9-EQR	Altered PAM	D1135E/R1335Q/T1337R	NGAG	
	Cas9-VRER	Altered PAM	D1135V/G1218R/R1335E/T1337R	NGCG	
	Cas9-VRQR	Altered PAM	M495V/Y515N/K526E/R661Q	NGA	
	Cas9-QQR1	Altered PAM	G1218R/N1286Q/I1331F/D1332K/R1333Q/R1335Q/T1337R	NAAG	(34)
	SpCas9-HF1	Reduced OTE	N497A/R661A/Q695A/Q926A	NGG	(35)
	eSpCas9	Reduced OTE	K846A/K1003A/R1060A	NGG	(36)
	HeFSpCas9	Reduced OTE	N497A/R661A/Q695A/K846A/Q926A/K1003A/R1060A	NGG	(37)
	evoCas9	Reduced OTE	M495V/Y515N/K526E/R661Q	NGG	(38)
	HiFiCas9	Reduced OTE	R691A	NGG	(39)
	Cas9n/Cas9D10A	SSB instead of DSB, Reduced OTE	D10A	NGG	(40, 41)
	Dimeric dCas9-FokI	Reduced OTE	dCas9 fused to FokI endonuclease domain	NGG	(42)
	xCas9-3.7	Broad PAM specificity	A262T/R324L/S409I/E480K/E543D/M694I/E1219V	NG, GAA or GAT	(43)
	SpCas9-NG	Minimal PAM	R1335V/L1111R/D1135V/G1218R/E1219F/A1322R/T1337R	NGN	(44)
	HypaCas9	Reduced OTE	N692A/M694A/Q695A/H698A	NGG	(45)
	Sniper-Cas9	Reduced OTE	F539S/M763I/K890N	NGG	(46)
	SpG Cas9	Minimal PAM	D1135L/S1136W/G1218K/E1219Q/R1335Q/T1337R	NGN	(47)
	SpRY Cas9	Minimal PAM	D1135L/S1136W/G1218K/E1219Q/R1335Q/T1337R/L1111R/A1322R/A61R/N1317R/R1333P	NRN>NYN	
	SpCas9-HF1 *plus*	Reduced OTE	N497A/Q695A/Q926A; amino acids 1005-1013 replaced with two glycine	NGG	(48)
	eSpCas9 *plus*	Reduced OTE	K848A/R1060A; amino acids 1005-1013 replaced with two glycine	NGG	
	Cas9_R63A/Q768A	Reduced OTE	R63A/Q768A	NGG	(49)
*Staphylococcus aureus*	KKH SaCas9	Relaxed PAM	E782K/N968K/R1015H	NNNRRT	(33)
	SaCas9-HF	Reduced OTE	R245A/N413A/N419A/R654A	NNGRRT	(50)
	SaCas9-NR	Relaxed PAM	N986R	NNGRR	(51)
	SaCas9-RL	Relaxed PAM	N986R/R991L	NNGRR	
*Streptococcus canis*	ScCas9	Minimal PAM	N/A (wildtype)	NNG	(52)

Optimizing guide designs can also reduce the frequency of OTEs (31). Many features in an sgRNA determine specificity including the seed sequence (a 10–12 bp region proximal to PAM on 3′ of spacer sequence) (29, 53), GC content (54, 55), and modifications such as 5′ truncation of the sgRNA (56). Several platforms have also been designed to provide optimized guide sequences against target genes, including E-Crisp (31, 57), CRISPR-design, CasOFFinder, and others (31). However, many of these tools are designed based on computational algorithms with varying parameters or rely on phenotypic screens that may be specific to cell types and genomes, generating appreciable noise and lack of generalizability across different experimental setups (58, 59). Recently, an additional guide design tool named sgDesigner was developed that addressed these limitations by employing a novel plasmid library *in silico* that contained both the sgRNA and the target site within the same construct. This allowed collecting Cas9 editing efficiency data in an intrinsic manner and establish a new training dataset that avoids the biases introduced through other models. Furthermore, a comparative performance evaluation to predict sgRNA efficiency of sgDesigner with 3 other commonly used tools (Doench Rule Set 2, Sequence Scan for CRISPR and DeepCRISPR) revealed that sgDesigner outperformed all 3 designer tools in 6 independent datasets, suggesting that sgDesigner may be a more robust and generalizable platform (60).

### Protospacer Adjacent Motif Requirement

An additional limitation of the technology is the requirement for a PAM near the target site. Cas9 from the bacteria *Streptococcus pyogenes* (SpCas9) is one of the most extensively used Cas9s with a relatively short canonical PAM recognition site: 5′NGG3′, where N is any nucleotide. However, SpCas9 is relatively large and difficult to package into AAV vectors (61, 62), the most common delivery vehicle for gene therapy. *Staphylococcus aureus* Cas9 (SaCas9) is a smaller ortholog that can be packaged more easily in AAV vectors but has a longer PAM sequence: 5′NNGRRT3′ or 5′NNGRR(N)3′, where R is any purine, which further narrows the window of therapeutic targeting sites. Engineered SaCas9 variants have been made, such as KKH SaCas9, which recognizes a 5′NNNRRT3′ PAM, broadening the human targeting sites by 2- to 4-fold. OTEs, however, are observed with frequencies similar to wildtype SaCas9 and need to be considered in designing any therapeutic application (33). Several other variants of SpCas9 have also been engineered for broadening the gene target window including SpCas9-NG, which recognizes a minimal NG PAM (44) and xCas9, which recognizes a broad range of PAM including NG, GAA, and GAT (43). A side by side comparison of both variants revealed that while SpCas9-NG had a broader PAM recognition, xCas9 had the lowest OTE in human cells (63). Another Cas9 ortholog from the bacteria *Streptococcus canis*, ScCas9, has been recently characterized with a minimal PAM specificity of 5′NNG3′ and an 89.2% sequence homology to SpCas9 and comparable editing efficiency to SpCas9 in both bacterial and human cells (52). The most recent development is a variant of SpCas9 named SpRY that has been engineered to be nearly PAMless, recognizing minimal NRN > NYN PAMs. This new variant can potentially edit any gene independent of a PAM requirement, and hence can be used therapeutically against several genetic diseases (47).

Alternatively, RNA-targeting Cas9 variants have been developed which also broaden the gene targeting spectrum by mitigating PAM requirement restrictions. *S. pyogenese* Cas9 (SpyCas9) can be manipulated to target RNA by providing a short oligonucleotide with a PAM sequence, known as a PAMmer (64, 65), and thus eliminates the need for a PAM site within the target region. Other subsets of Cas enzymes have also been discovered that naturally target RNA independent of a PAM, such as Cas13d. Upon further engineering of this effector, CasRx was developed for efficient RNA-guided RNA targeting in human cells (66, 67). Although RNA-targeting CRISPR advances provide a therapeutic opportunity without the risk of DNA-damage toxicity, they exclude the potential for editing a permanent correction into the genome.

### DNA-Damage Toxicity

CRISPR-induced DSBs often trigger apoptosis rather than the intended gene edit (68). Further safety concerns were revealed when using this tool in human pluripotent stem cells (hPSCs) which demonstrated that p53 activation in response to the toxic DSBs introduced by CRISPR often triggers subsequent apoptosis (69). Thus, successful CRISPR edits are more likely to occur in p53 suppressed cells, resulting in a bias toward selection for oncogenic cell survival (70). In addition, large deletions spanning kilobases and complex rearrangements as unintended consequences of on-target activity have been reported in several instances (71, 72), highlighting a major safety issue for clinical applications of DSB-inducing CRISPR therapy. Other variations of Cas9, such as catalytically inactive endonuclease dead Cas9 (dCas9) in which the nuclease domains are deactivated, may provide therapeutic utility while mitigating the risks of DSBs (73). dCas9 can transiently manipulate expression of specific genes without introducing DSBs through fusion of transcriptional activating or repressing domains or proteins to the DNA-binding effector (74). Other variants such as Cas9n can also be considered, which induces SSBs rather than DSBs. Further modifications of these Cas9 variants has led to the development of base editors and prime editors, a key innovation for safe therapeutic application of CRISPR technology (see Precision Gene Editing With CRISPR section).

### Immunotoxicity

In addition to technical limitations, CRISPR/Cas9, like traditional gene therapy, still raises concerns for immunogenic toxicity. Charlesworth et al. showed that more than half of the human subjects in their study possessed preexisting anti-Cas9 antibodies against the most commonly used bacterial orthologs, SaCas9 and SpCas9 (75). Furthermore, AAV vectors are also widely used to deliver CRISPR components for gene therapy. To this end, several Cas9 orthologs and AAV serotypes were tested based on sequence similarities and predicted binding strength to MHC class I and class II to screen for immune orthologs that can be used for safe repeated administration of AAV-CRISPR gene therapy. Although no two AAV serotypes were found to completely circumvent immune recognition, the study verified 3 Cas9 orthologs [SpCas9, SaCas9, and *Campylobacter jejuni* Cas9 (CjCas9)] which showed robust editing efficiency and tolerated repeated administration due to reduced immunogenic toxicity in mice immunized against AAV and Cas9 (76). A major caveat is pre-existing immunity in humans against 2 of these orthologs—SpCas9 and SaCas9, leaving CjCas9 as the only current option for this cohort of patients. However, this ortholog has not been well-studied in comparison to the other 2 orthologs and will need further investigation to provide evidence for its safety and efficacy for clinical use. Future studies may also identify other Cas9 immune-orthogonal orthologs for safe repeated gene therapy.

### Precision Gene Editing With CRISPR

Precise-genome editing is essential for prospects of CRISPR gene therapy. Although HDR pathways can facilitate a desired edit, its low efficiency renders its utility for precise gene editing for clinical intervention highly limiting, with NHEJ as the default pathway human cells take for repair. Enhancement of HDR efficiency has been achieved via suppression of the NHEJ pathway through chemical inhibition of key NHEJ modulating enzymes such as Ku (77), DNA ligase IV (78), and DNA-dependent protein kinases (DNA-PKcs) (79). Other strategies that improve HDR efficiency include using single-stranded oligodeoxynucleotide (ssODN) template, which contains the homology arms to facilitate recombination and the desired edit sequence, instead of double-stranded DNA (dsDNA). Rationally designed ssODN templates with optimized length complementarity have been shown to increase HDR rates up to 60% in human cells for single nucleotide substitution (80). Furthermore, cell cycle stage plays a key role in determining the DNA-damage repair pathway a cell may take. HDR events are generally restricted to late S and G2 phases of the cell cycle, given the availability of the sister chromatid to serve as a template at these stages, whereas NHEJ predominates the G1, S, and G2 phases (81). Pharmacological arrest at the S phase with aphidicolin increased HDR frequency in HEK293T with Cas9-guide ribonucleoprotein (RNP) delivery. Interestingly, cell arrest in the M phase using nocodazole with low concentrations of the Cas9-guide RNP complex yielded higher frequencies of HDR events in these cells, reaching a maximum frequency of up to 31% (82). Although HDR is considered to be restricted to mitotic cells, a recent study revealed that the CRISPR/Cas9 editing can achieve HDR in mature postmitotic neurons. Nishiyama et al. successfully edited the CaMKIIα locus through HDR in postmitotic hippocampal neurons of adult mice *in vitro* using an AAV delivered Cas9, guide RNA, and donor template in the CaMKIIα locus, which achieved successful HDR-mediated edits in ~30% of infected cells. Although HDR efficiency was dose-dependent on AAV delivered HDR machinery and off-target activity was not monitored, this study demonstrated CRISPR's potential utility for translational neuroscience after further developments (83). To further exploit cell-cycle stage control as a means to favor templated repair, Cas9 conjugation to a part of Geminin, a substrate for G1 proteosome degradation, can limit Cas9 expression to S, G2, and M stages. This strategy was shown to facilitate HDR events while mitigating undesired NHEJ edits in human immortalized and stem cells (84, 85). A more recent strategy combined a chemically modified Cas9 to the ssODN donor or a DNA adaptor that recruits the donor template, either of which improved HDR efficiency by localizing the donor template near the cleavage site (86). Despite these advancements, HDR is still achieved at a relatively low efficiency in eukaryotic cells and use of relatively harmful agents in cells such as NHEJ chemical inhibitors may not be ideal in a clinical setting.

**Figure 3:**
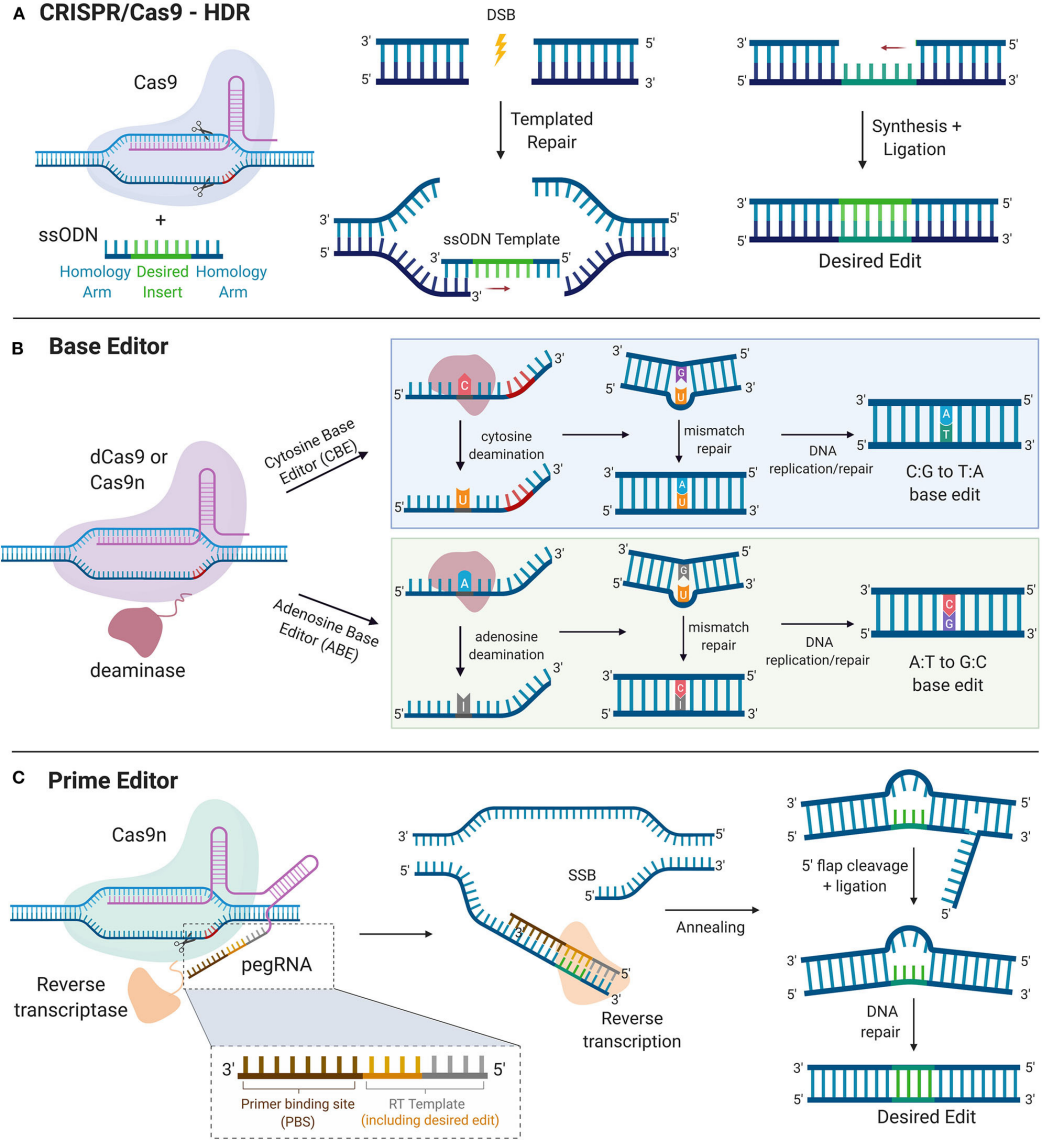
Precise Gene Editing. **(A)** CRISPR/Cas9-HDR. Cas9 induces a DSB. The exogenous ssODN carrying the sequence for the desired edit and homology arms is used as a template for HDR-mediated gene modification. **(B)** Base Editor. dCas9 or Cas9n is tethered to the catalytic portion of a deaminase. Cytosine deaminase catalyzes the formation of uridine from cytosine. DNA mismatch repair mechanisms or DNA replication yield an C:G to T:A single nucleotide base edit. Adenosine deaminase catalyzes the formation of inosine from adenosine. DNA mismatch repair mechanisms or DNA replication yield an A:T to G:C single nucleotide base edit. **(C)** Prime Editor. Cas9n is tethered to the catalytic portion of reverse transcriptase. The prime editor system uses pegRNA, which contains the guide spacer sequence, reverse transcriptase primer, which includes the sequence for the desired edit and a primer binding site (PBS). PBS hybridizes with the complementary region of the DNA and reverse transcriptase transcribes new DNA carrying the desired edit. After cleavage of the resultant 5′ flap and ligation, DNA repair mechanisms correct the unedited strand to match the edited strand. HDR, homology directed repair. DSB, double stranded break; SSB, single-stranded break; ssODN, single-stranded oligodeoxynucleotide.

A recent advancement that allows precision gene editing independent of exploiting DNA damage response mechanisms is the CRISPR base editing (BE) system. In this system, a catalytically inactive dead Cas9 (dCas9) is conjugated to deaminase, which can catalyze the conversion of nucleotides via deamination. For increased editing efficiency, Cas9 nickase (Cas9n) fused with deaminase is recently being more utilized over dCas9 for base editing, as the nicks created in a single strand of DNA induce higher editing efficiency. Currently, the two types of CRISPR base editors are cytidine base editors (CBEs) and adenosine base editors (ABEs). CBEs catalyze the conversion of cytidine to uridine, which becomes thymine after DNA replication. ABEs catalyze the conversion of adenosine to inosine which becomes guanine after DNA replication (87). Base editors provide a means to edit single nucleotides without running the risk of causing DSB-induced toxicity. However, base editors are limited to “A to T” and “C to G” conversions, narrowing its scope for single-base gene edition to only these bases. In addition, base editors still face some of the same challenges as the previously described CRISPR systems, including OTEs, more so with CBEs than ABEs (88, 89) and packaging constraints, namely in AAV vectors due to the large size of base editors (90). Furthermore, the editing window for base editors are limited to a narrow range of a few bases upstream of the PAM (90). More recently, prime editing has been developed as a strategy to edit the genome to insert a desired stretch of edits without inducing DSBs (91). This technology combines fusion of Cas9n with a reverse transcriptase and a prime editing guide RNA (pegRNA), which contains sgRNA sequence, primer binding site (PBS), and an RNA template encoding the desired edit on the 3′ end. Prime editors use Cas9n to nick one strand of the DNA and insert the desired edit via reverse transcription of the RNA template. The synthesized edit is incorporated into the genome and the unedited strand is cleaved and repaired to match the inserted edit. With an optimized delivery system in place, base editors and primer editors can open the door for precision gene editing to correct and potentially cure a multitude of genetic diseases (Figure 3).

### Delivery of CRISPR Gene Therapy

The delivery modality of CRISPR tools greatly influences its safety and therapeutic efficacy. While traditional gene therapy utilizing viruses have been scrutinized for the risk of immunotoxicity and insertional oncogenesis, AAV vectors remain a key delivery vehicle for CRISPR gene therapy and continues to be extensively used for its high efficiency of delivery (92). The CRISPR toolkit can be packaged as plasmid DNA encoding its components, including Cas9 and gRNA, or can be delivered as mRNA of Cas9 and gRNA. Nucleic acids of CRISPR can be packaged in AAV vectors for delivery or introduced to target cells via electroporation/nucleofection or microinjection, with the latter methods averting virus-associated risks. However, microinjection can be technically challenging and is only suited for *ex vivo* delivery. Electroporation is also largely used for *ex vivo* but can be used *in vivo* for certain target tissues (93). However, high-voltage shock needed to permeabilize cell membranes via electroporation can be toxic and can lead to permanent permeabilization of treated cells (94). In addition to viral toxicity, AAV delivery of CRISPR components yields longevity of expression, leading to greater incidence of OTEs. Alternatively, delivery of the Cas9 protein and gRNA as RNP complexes has reduced OTEs while maintained editing efficacy, owing to its transient expression and rapid clearance in the cell (95).

**Figure 4:**
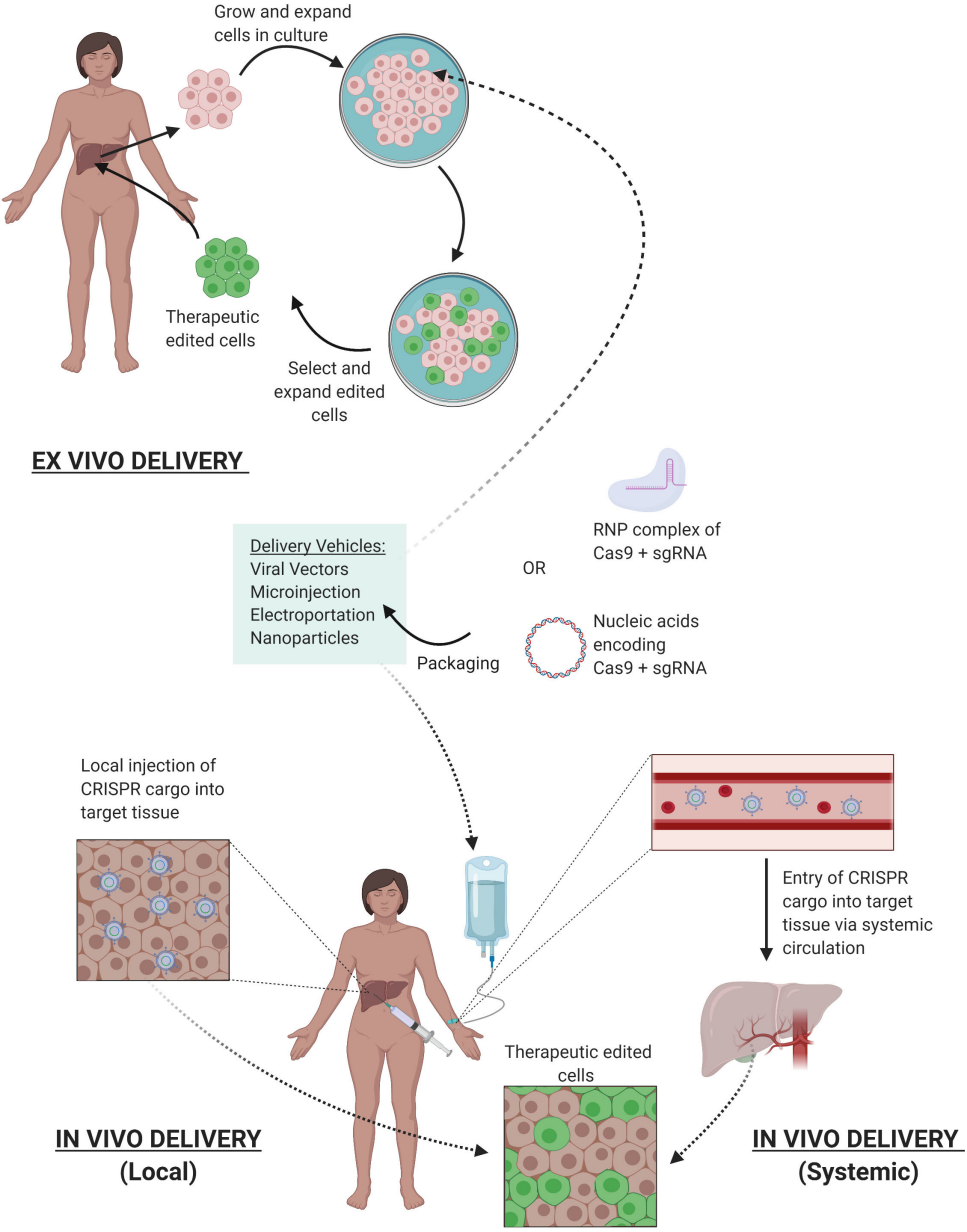
Delivery of CRISPR Therapy. Nucleic acids encoding CRISPR/Cas9 or its RNP complex can be packaged into delivery vehicles. Once packaged, edits can be facilitated either *ex vivo* or *in vivo*. *Ex vivo* editing involves extraction of target cells from the patient, cell culture, and expansion *in vitro*, delivery of the CRISPR components to yield the desired edits, selection, and expansion of edited cells, and finally reintroduction of therapeutic edited cells into the patient. *In vivo* editing can be systemically delivered via intravenous infusions to the patient, where the CRISPR cargo travels through the bloodstream via arteries leading to the target tissue, or locally delivered with injections directly to target tissue. Once delivered, the edits are facilitated *in vivo* to provide therapeutic benefit.

Once the delivery modality is selected, CRISPR/Cas9 edits can be facilitated either *ex vivo* where cells are genetically modified outside of the patient and reintroduced back, or *in vivo* with delivery of the CRISPR components directly into the patient where cells are edited (Figure 4). Both systems pose their own set of advantages and challenges. Advantages for *ex vivo* delivery include greater safety since patients are not exposed to the gene altering tool, technical feasibility, and tighter quality control of the edited cells. However, challenges to this method include survival and retention of *in vivo* function of cells outside the patient after genetic manipulation and extensive culture *in vitro*. Also, an adequate supply of cells is needed for efficient re-engraftment. These conditions limit this method to certain cell types that can survive and be expanded in culture, such as hematopoietic stem and progenitor cells (HSPCs) (96) and T cells (97).

While *ex vivo* gene therapy has provided therapeutic benefit for hematological disorders and cancer immunotherapy, many tissue types are not suited for this method, severely limiting its therapeutic utility for other genetic diseases. *in vivo* manipulation is thus needed to expand CRISPR's utility to treat a broader range of genetic diseases, such as Duchenne muscular dystrophy (DMD) (98) and hereditary tyrosinemia (99). CRISPR components can be delivered *in vivo* systemically through intravenous injections or can be locally injected to specific tissues (Figure 4). With systemic delivery, the CRISPR components and its vehicle are introduced into the circulatory system where expression of the gene editing toolkit can be controlled to target specific organs via tissue-specific promoters (100). However, challenges of *in vivo* delivery include degradation by circulating proteases or nucleases, opsonization by opsonins, or clearance by the mononuclear phagocyte system (MPS). Furthermore, the cargo must reach the target tissue and bypass the vascular endothelium, which are often tightly connected by cell-cell junctions (101), preventing accessibility to larger delivery vehicles (>1 nm diameter). Additionally, once the cargo has reached the target cells, they must be internalized, which is generally facilitated through endocytosis where they can be transported and degraded by lysosomal enzymes (102). In addition, localization of the editing machinery near the point of injection can result in uneven distribution of the edited cell repertoire within the tissue, which may result in suboptimal therapeutic outcomes (102). While advancements are continuing to refine delivery techniques, the current systems have allowed CRISPR gene therapy to be used in the clinic.

## Biological Intervention of CRISPR/Cas9 in Clinical Trials

### Cancer Immunotherapy

The first CRISPR Phase 1 clinical trial in the US opened in 2018 with the intent to use CRISPR/Cas9 to edit autologous T cells for cancer immunotherapy against several cancers with relapsed tumors and no further curative treatment options. These include multiple myeloma, melanoma, synovial sarcoma and myxoid/round cell liposarcoma. This trial was approved by the United States Food and Drug Administration (FDA) after careful consideration of the risk to benefit ratios of this first application of CRISPR gene therapy into the clinic. During this trial, T lymphocytes were collected from the patients' blood and *ex vivo* engineered with CRISPR/Cas9 to knockout the α and β chains of the endogenous T cell receptor (TCR), which recognizes a specific antigen to mediate an immune response, and the programmed cell death-1 (PD-1) protein, which attenuates immune response. The cells were then transduced with lentivirus to deliver a gene encoding a TCR specific for a NY-ESO-1 antigen, which has been shown to be highly upregulated in the relapsed tumors and thus can serve as a therapeutic target. Since then, many trials have opened for CRISPR-mediated cancer immunotherapy and is currently the most employed strategy for CRISPR gene therapy (Table 2). A trial implementing this strategy using other tools had already been conducted in both pre-clinical and clinical settings, but this was the first time CRISPR/Cas9 was used to generate the genetically modified T cells (97). The moderate transition of switching only the tool used for an already approved therapeutic strategy may have been key to paving the road for using CRISPR's novel abilities for gene manipulation, such as targeted gene disruption.

**Table 2:** Biological intervention of CRISPR gene therapy in clinical trials.

**Sponsor/affiliation**	**Disease**	**Gene target**	**Clinial Trial ID**	**CRISPR-Cas9 mediated intervention**
University of Pennsylvania/Parker Institute for Cancer Immunotherapy/Tmunity	Multiple Myeloma, Melanoma, Synovial Sarcoma, Myxoid/Round Cell Liposarcoma	TCRα, TCRβ, PDCD1	NCT03399448	NY-ESO-1 redirected autologous T cells with CRISPR edited endogenous TCR and PD-1
Affiliated Hospital to Academy of Military Medical Sciences/Peking University/Capital Medical University	HIV-1	CCR5	NCT03164135	CD34+ hematopoietic stem/progenitor cells from donor are treated with CRISPR/Cas9 targeting CCR5 gene
CRISPR Therapeutics AG	Multiple Myeloma	TCRα, TCRβ, B2M	NCT04244656	CTX120 B-cell maturation antigen (BCMA)-directed T-cell immunotherapy comprised of allogeneic T cells genetically modified *ex vivo* using CRISPR-Cas9 gene editing components
Crispr Therapeutics/Vertex	Beta-Thalassemia, Thalassemia, Genetic Diseases Inborn, Hematologic Diseases, Hemoglobinopathies	BCL11A	NCT03655678	CTX001 (autologous CD34+ hHSPCs modified with CRISPR-Cas9 at the erythroid lineage-specific enhancer of the BCL11A gene)
Crispr Therapeutics	B-cell MalignancyNon-Hodgkin LymphomaB-cell Lymphoma	TCRα, TCRβ	NCT04035434	CTX110 (CD19-directed T-cell immunotherapy comprised of allogeneic T cells genetically modified *ex vivo* using CRISPR-Cas9 gene editing components)
Editas Medicine, Inc./Allergan	Leber Congenital Amaurosis 10	CEP290	NCT03872479	Single escalating doses of AGN-151587 (EDIT-101) administered via subretinal injection
Vertex Pharmaceuticals Incorporated/CRISPR Therapeutics	Sickle Cell Disease, Hematological Diseases, Hemoglobinopathies	BCL11A	NCT03745287	CTX001 (autologous CD34+ hHSPCs modified with CRISPR-Cas9 at the erythroid lineage-specific enhancer of the BCL11A gene)
Allife Medical Science and Technology Co., Ltd.	Thalassemia	HBB	NCT03728322	Investigate the safety and efficacy of the gene correction of HBB in patient-specific iHSCs using CRISPR/Cas9
Yang Yang, The Affiliated Nanjing Drum Tower Hospital of Nanjing University Medical School	Stage IV Gastric Carcinoma, Stage IV Nasopharyngeal Carcinoma, T-Cell Lymphoma Stage IV, Stage IV Adult Hodgkin Lymphoma, Stage IV Diffuse Large B-Cell Lymphoma	PDCD1	NCT03044743	CRISPR-Cas9 mediated PD-1 knockout-T cells from autologous origin
First Affiliated Hospital, Sun Yat-Sen University/Jingchu University of Technology	Human Papillomavirus-Related Malignant Neoplasm	HPV16 and HPV18 E6/E7 DNA	NCT03057912	Evaluate the safety and efficacy of TALEN-HPV E6/E7 and CRISPR/Cas9-HPV E6/E7 in treating HPV Persistency and HPV-related Cervical Intraepithelial NeoplasiaI
Sichuan University/Chengdu MedGenCell, Co., Ltd.	Metastatic Non-small Cell Lung Cancer	PDCD1	NCT02793856	CRISPR-Cas9 mediated PD-1 knockout-T cells from autologous origin
Peking University	Metastatic Renal Cell Carcinoma	PDCD1	NCT02867332	CRISPR-Cas9 mediated PD-1 knockout-T cells from autologous origin
Peking University	Hormone Refractory Prostate Cancer	PDCD1	NCT02867345	CRISPR-Cas9 mediated PD-1 knockout-T cells from autologous origin
Peking University	Invasive Bladder Cancer Stage IV	PDCD1	NCT02863913	CRISPR-Cas9 mediated PD-1 knockout-T cells from autologous origin
Hangzhou Cancer Hospital/Anhui Kedgene Biotechnology Co., Ltd	Esophageal Cancer	PDCD1	NCT03081715	CRISPR-Cas9 mediated PD-1 knockout-T cells from autologous origin
Chinese PLA General Hospital	Solid Tumor, Adult	TCRα, TCRβ, PDCD1	NCT03545815	Evaluate the feasibility and safety of CRISPR-Cas9 mediated PD-1 and TCR gene-knocked out chimeric antigen receptor (CAR) T cells in patients with mesothelin positive multiple solid tumors
Baylor College of Medicine/The Methodist Hospital System	T-cell Acute Lymphoblastic Leukemia, T-cell Acute Lymphoblastic Lymphoma, T-non-Hodgkin Lymphoma	CD7	NCT03690011	CRISPR-Cas9 mediated CD7 knockout-T cells from autologous origin
Chinese PLA General Hospital	B Cell Leukemia, B Cell Lymphoma	PDCD1	NCT03398967	Determine the safety of the allogenic CRISPR-Cas9 gene-edited dual specificity CD19 and CD20 or CD22 CAR-T cells
Chinese PLA General Hospital	B Cell Leukemia, B Cell Lymphoma	TCRα, TCRβ, B2M	NCT03166878	CRISPR-Cas9 mediated TCR and B2M knockout-T cells from allogenic origin for CD19 CAR-T
Chinese PLA General Hospital	Solid Tumor, Adult	PDCD1	NCT03747965	CRISPR-Cas9 mediated PD-1 knockout-T cells from autologous origin
Xijing Hospital/Xi'An Yufan Biotechnology Co., Ltd	Leukemia, Lymphoma	HPK1	NCT04037566	CRISPR Gene Edited to Eliminate Endogenous HPK1 (XYF19 CAR-T Cells)

### Gene Disruption

The first clinical trial in the US using CRISPR to catalyze gene disruption for therapeutic benefit were for patients with sickle-cell anemia (SCD) and later β-thalassemia, by Vertex Pharmaceuticals and CRISPR Therapeutics. This therapy, named CTX001, increases fetal hemoglobin (HbF) levels, which can occupy one or two of four hemoglobin binding pockets on erythrocytes and thereby provides clinical benefit for major β-hemoglobin diseases such as SCD and β-thalassemia (103). The trial involved collecting autologous hematopoietic stem and progenitor cells from peripheral blood and using CRISPR/Cas9 to disrupt the intronic erythroid-specific enhancer for the *BCL11A* gene (NCT03745287) as disruption of this gene increases HbF expression (104–106). Genetically modified hematopoietic stem cells with *BCL11A* disruption are delivered by IV infusion after myeloablative conditioning with busulfan to destroy unedited hematopoietic stem cells in the bone marrow. Preliminary findings from two patients receiving this treatment seem promising. One SCD patient was reported to have 46.6% HbF and 94.7% erythrocytes expressing HbF after 4 months of CTX001 transfusions and one β-thalassemia patient is expressing 10.1 g/dL HbF out of 11.9 g/dL total hemoglobin, and 99.8% erythrocytes expressing HbF after 9 months of the therapy. Results from the clinical trial that has opened for this therapy (NCT04208529) to assess the long-term risks and benefits of CTX001 will dictate whether this approach can provide a novel therapeutic opportunity for a disease that otherwise has limited treatment options.

### *In vivo* CRISPR Gene Therapy

While the aforementioned trials rely on *ex vivo* editing and subsequent therapy with modified cells, *in vivo* approaches have been less extensively employed. An exciting step forward with CRISPR gene therapy has been recently launched with a clinical trial using *in vivo* delivery of CRISPR/Cas9 for the first time in patients. While *in vivo* editing has been largely limited by inadequate accessibility to the target tissue, a few organs, such as the eye, are accessible. Leber congenital amaurosis (LCA) is a debilitating monogenic disease that results in childhood blindness caused by a bi-allelic loss-of-function mutation in the *CEP290* gene, with no treatment options. This therapy, named EDIT-101, delivers CRISPR/Cas9 directly into the retina of LCA patients specifically with the intronic IVS26 mutation, which drives aberrant splicing resulting in a non-functional protein. The therapy uses an AAV5 vector to deliver nucleic acid instructions for *Staphylococcus aureus* Cas9 and two guides targeting the ends of the *CEP290* locus containing the IVS26 mutation. The DSB induced by Cas9 and both guides result in either a deletion or inversion of the IVS26 intronic region, thus preventing the aberrant splicing caused by the genetic mutation and enabling subsequent translation of the functional protein (107). Potential immunotoxicity or OTEs arising from nucleic acid viral delivery will have to be closely monitored. Nonetheless, a possibly curative medicine for genetic blindness using an *in vivo* approach marks an important advancement for CRISPR gene therapy.

### CRISPR Editing in Human Embryos and Ethical Considerations

While somatic editing for CRISPR therapy has been permitted after careful consideration, human germline editing for therapeutic intent remains highly controversial. With somatic edition, any potential risk would be contained within the individual after informed consent to partake in the therapy. Embryonic editing not only removes autonomy in the decision-making process of the later born individuals, but also allows unforeseen and permanent side effects to pass down through generations. This very power warrants proceeding with caution to prevent major setbacks as witnessed by traditional gene therapy. However, a controversial CRISPR trial in human embryos led by Jiankui He may have already breached the ethical standards set in place for such trials. This pilot study involved genetic engineering of the C-C chemokine receptor type 5 (*CCR5*) gene in human embryos, with the intention of conferring HIV-resistance, as seen by a naturally occurring *CCR5*Δ*32* mutation in a few individuals (108). However, based on the limited evidence, CRISPR/Cas9 was likely used to target this gene, but rather than replicate the naturally observed and beneficial 32-base deletion, the edits merely induced DSBs at one end of the deletion, allowing NHEJ to repair the damaged DNA while introducing random, uncharacterized mutations. Thus, it is unknown whether the resultant protein will function similarly to the naturally occurring *CCR5*Δ*32* protein and confer HIV resistance. In addition, only one of the two embryos, termed with the pseudonym Nana, had successful edits in both copies of the *CCR5* gene, whereas the other embryo, with pseudonym Lulu, had successful editing in only one copy. Despite these findings, both embryos were implanted back into their mother, knowing that the HIV-resistance will be questionable in Nana and non-existent in Lulu (109, 110).

Furthermore, recent studies have shown that the mechanism for infection of some variants of the highly mutable HIV virus may heavily rely on the C-X-C chemokine receptor type 4 (*CXCR4*) co-receptor (108, 111). With no attempts at editing *CXCR4*, this adds yet another layer of skepticism toward achieving HIV resistance by this strategy. In addition, OTEs, particularly over the lifetime of an individual, remain a major concern for applying this technology in humans. The recent advances in the editing tool to limit OTEs, such as using high fidelity Cas9 variants, has not been exploited. Furthermore, the rationale for selecting HIV prevention for the first use of CRISPR in implanted human embryos contributes to the poor risk to benefit ratio of this study, considering HIV patients can live long, healthy lives on a drug regimen. A more appropriate first attempt would have been to employ this technology for a more severe disease. For example, correction of the *MYBPC3* gene is arguably a better target for embryonic gene editing, as mutations in *MYBPC3* can cause hypertrophic cardiomyopathy (HCM), a heart condition responsible for most sudden cardiac deaths in people under the age of 30. Gene correction for this pathological mutation was achieved recently for the first time in the US in viable human embryos using the HDR-mediated CRISPR/Cas9 system. However, these embryos were edited for basic research purposes and not intended for implantation. In this study, sperm carrying the pathogenic *MYBPC3* mutation and the CRISPR/Cas9 machinery as an RNP complex were microinjected into healthy donor oocytes arrested at MII, achieving 72.4% homozygous wildtype embryos as opposed to 47.4% in untreated embryos. The HDR-mediated gene correction was observed at considerably high frequencies with no detectable OTEs in selected blastomeres, likely owing to the direct microinjection delivery of the RNP complex in the early zygote. Interestingly, the maternal wildtype DNA was used preferentially for templated repair over the provided exogenous ssODN template (112). While evidence for gene correction was promising, NHEJ mediated DNA repair was still observed in many embryos, highlighting the need to improve HDR efficiency before clinical application can be considered. Although strategies have been developed to improve HDR, such as chemical inhibitors of NHEJ (77–79), such techniques may have varying outcomes in embryonic cells and side effects that may arise from treatment needs to be investigated. Germline gene editing will remain to be ethically unfavorable at its current state and its discussions may not be considered until sufficient long-term studies of the ongoing somatic CRISPR therapy clinical trials are evaluated.

## Potential for CRISPR Therapeutics During COVID-19 Pandemic

The rapidly advancing CRISPR technology may provide aid during our rapidly evolving times. The recent outbreak of a novel severe acute respiratory syndrome coronavirus 2 (SARS-CoV-2) has led to a global pandemic (113). These pressing times call for an urgent response to develop quick and efficient testing tools and treatment options for coronavirus disease 2019 (COVID-19) patients. Currently available methods for testing are relatively time consuming with suboptimal accuracy and sensitivity (114). The two predominant testing methods are molecular testing or serological testing. The US Centers for Disease Control and Prevention (CDC) has developed a real-time RT-PCR assay for molecular testing for the presence of viral RNA to detect COVID-19 (115). However, this assay has a roughly ~30% false negative rate (116, 117) with the turnaround time of several hours to >24 h. Serological testing methods are much more rapid but lack the ability to detect acute respiratory infection since antibodies used to detect infection can take several days or weeks to develop.

Recently, a CRISPR Cas12-based assay named SARS-CoV-2 DETECTR has been developed for detection of COVID-19 with a short turnaround time of about 40 min and a 95% reported accuracy. The assay involves RNA extraction followed by reverse transcription and simultaneous isothermal amplification using the RT-LAMP method. Cas12 and a guide RNA against regions of the N (nucleoprotein) gene and E (envelope) gene of SARS-CoV-2 are then targeted, which can be visualized by cleavage of a fluorescent reporter molecule. The assay also includes a laminar flow strip for a visual readout, where a single band close to where the sample was applied indicates a negative test and 2 higher bands or a single higher band would indicate cleavage of the fluorescent probe and hence positive for SARS-CoV-2 (118).

In addition to CRISPR's diagnostic utility, CRISPR may provide therapeutic options for COVID-19 patients. The recently discovered Cas13 is an RNA-guided RNA-targeting endonuclease may serve as a potential therapeutic tool against COVID-19. PAC-MAN (Prophylactic Antiviral CRISPR in huMAN cells) has been developed, which utilizes the *Ruminococcus flavefaciens* derived VI-D CRISPR-Cas13d variant, selected for its small size facilitating easier packaging in viral vehicles, high specificity, and strong catalytic activity in human cells. This technique was developed to simultaneously target multiple regions for RNA degradation, opening the door for a much-needed pan-coronavirus targeting strategy, given the evidence suggesting relatively high mutation and recombination rates of SARS-CoV-2 (119). With these advances, the CRISPR/Cas machinery may again be implemented to serve its original purpose as a virus-battling system to provide aid during this pandemic.

## Discussion

The birth of gene therapy as a therapeutic avenue began with the repurposing of viruses for transgene delivery to patients with genetic diseases. Gene therapy enjoyed an initial phase of excitement, until the recognition of immediate and delayed adverse effects resulted in death and caused a major setback. More recently, the discovery and development of CRISPR/Cas9 has re-opened a door for gene therapy and changed the way scientists can approach a genetic aberration—by fixing a non-functional gene rather than replacing it entirely, or by disrupting an aberrant pathogenic gene. CRISPR/Cas9 provides extensive opportunities for programmable gene editing and can become a powerful asset for modern medicine. However, lessons learned from traditional gene therapy should prompt greater caution in moving forward with CRISPR systems to avoid adverse events and setbacks to the development of what may be a unique clinically beneficial technology. A failure to take these lessons into account may provoke further backlash against CRISPR/Cas9 development and slow down progression toward attaining potentially curative gene editing technologies.

Although CRISPR editing in humans remains a highly debated and controversial topic, a few Regulatory Affairs Certification (RAC)-reviewed and FDA-approved CRISPR gene therapy trials have opened after thorough consideration of the risk to benefit ratios. These first few approved trials, currently in Phase I/II, are only for patients with severe diseases, such as cancers or debilitating monogenic diseases. The outcomes of these trials will dictate how rapidly we consider using this system to treat less severe diseases, as the risks of the technology are better understood. A concern remains whether normalizing CRISPR/Cas9 editing for less debilitating diseases may act as a gateway for human genome editing for non-medical purposes, such as altering genes in embryos to create offspring with certain aesthetic traits. This fear of unnatural selection for unethical reasons has likely become more tangible in the public's view with the strong media attention of the edited “CRISPR babies.” The lasting effects of that trial and outcomes of the approved clinical trials will greatly influence CRISPR's future in gene therapy and begin to answer the key questions we must consider as we further explore this technology. These key questions include how to avoid the mistakes of the past, who should decide CRISPR's therapeutic future, and how the ethical boundaries of its applications should best be drawn.

## Author Contributions

FU researched and drafted the article. TS and CR supervised the content. All authors wrote, reviewed, and edited the manuscript before submission.

## Conflict of Interest

CR has consulted regarding oncology drug development with AbbVie, Amgen, Ascentage, Astra Zeneca, Celgene, Daiichi Sankyo, Genentech/Roche, Ipsen, Loxo, and Pharmar, and is on the scientific advisory boards of Harpoon Therapeutics and Bridge Medicines. The remaining authors declare that the research was conducted in the absence of any commercial or financial relationships that could be construed as a potential conflict of interest.
